# Peer review and the publication process

**DOI:** 10.1002/nop2.51

**Published:** 2016-03-16

**Authors:** Parveen Azam Ali, Roger Watson

**Affiliations:** ^1^ The School of Nursing and MidwiferyThe University of SheffieldBarber House Annexe3a Clarkehouse RoadSheffieldS10 2LAUK; ^2^ Faculty of Health and Social CareUniversity of HullCottingham RoadHullHU6 7RXUK

**Keywords:** Double blind peer review, manuscript, peer review, peer reviewer, publication process, single blind peer review

## Abstract

**Aims:**

To provide an overview of the peer review process, its various types, selection of peer reviewers, the purpose and significance of the peer review with regard to the assessment and management of quality of publications in academic journals.

**Design:**

Discussion paper.

**Methods:**

This paper draws on information gained from literature on the peer review process and the authors' knowledge and experience of contributing as peer reviewers and editors in the field of health care, including nursing.

**Results:**

There are various types of peer review: single blind; double blind; open; and post‐publication review. The role of the reviewers in reviewing manuscripts and their contribution to the scientific and academic community remains important.

## Introduction

Publication in academic journals plays an important role in the development and progress of any profession, including nursing (Dipboye 2006). On the one hand, it provides professionals such as nurses with an opportunity to share their examples of best practice and research results with colleagues in the discipline. On the other hand, academic and scientific publications serve as a source of knowledge and evidence for students, novice practitioners and emerging researchers (Henly & Dougherty 2009) and contribute to their professional development. To serve these purposes effectively, appropriate scrutiny of manuscripts submitted to academic journals, to determine their worth, quality, methodological rigour, utility and publishability before appearing in the electronic and print media, is warranted. Such quality assurance mechanisms are essential to ensure publication of reliable and high quality research and scholarly evidence (Shattell *et al*. 2010).

**Figure 1:**
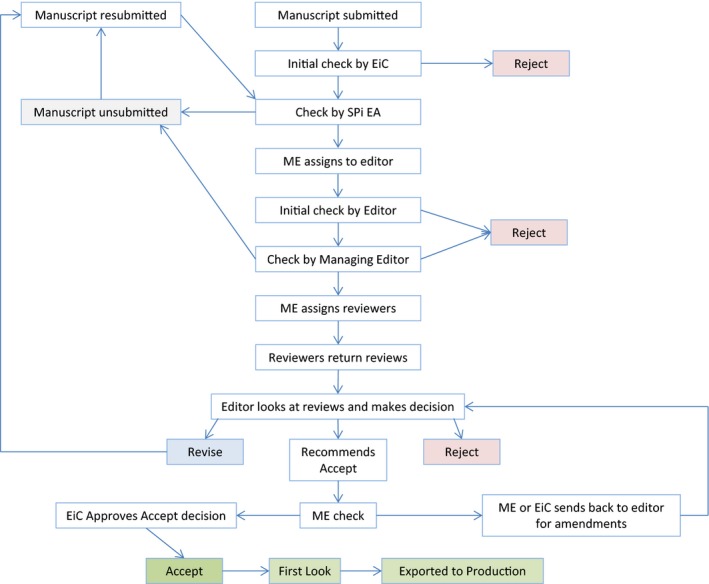
The editorial process, including peer review. EiC, editor‐in‐chief; EA, editorial assistant (SPi is a company providing editorial assistants); ME, managing editor.

The publication process begins with a manuscript submission to a journal by an author. As shown in Figure 1 – which outlines the editorial processes at Wiley – a manuscript goes through several stages before actual publication (Jefferson *et al*. 2007). The process outlined in Figure 1 may be more elaborate than for some journals and the various tasks may be distributed differently across the editorial team, but this figure includes all of the possible steps that can take place in the publication process. The first stage of the process is an editorial review that aims to assess the quality and merits of a manuscript. The editor (often the editor‐in‐chief) of the journal concerned reviews the manuscript to determine its relevance to the journal and suitability to undergo peer review. Further checks take place at the editorial desk by an editorial assistant, including checks for similarity to other sources using a similarity detection package such as iThenticate^®^. If the manuscript is too similar to other sources, it may be rejected or it may be unsubmitted and returned to the author for amendment. Additional checks for readability and the extent to which the manuscript conforms to the standards of the journal, for example, word‐length and use of international reporting standards take place. In Figure 1, this is done by a managing editor and, again, the manuscript may be rejected or returned to the author for amendment. Once satisfied, the managing editor assigns an editor, identifies, and assigns 2‐3 reviewers with appropriate knowledge, skills, methodological expertise and experience to assess the manuscript and feedback on its quality, rigour and publishability. Peer reviewers' feedback helps the editor to decide if the manuscript is rejected, accepted or needs revision before it can be accepted for publication. Whatever the case, the decision is communicated to the author. When a revision is required, the reviewers suggest changes or ask for more details from the authors before accepting the manuscript for publication. Once the manuscript is accepted, it moves to the third stage, which is called production and ensures the production of a readable and comprehensible article free of spelling mistakes, and presented in the uniform style of a particular journal (Jefferson *et al*. 2007). The author is also expected to check and approve the final proof before the final stage which is an administrative process, to ensure the allocation of appropriate tracking number, called Digital Object Identifier (DOI), to the article and regular production of a journal (Jefferson *et al*. 2007). The peer review process is important to understand, not only for potential authors, but also for those involved in the process, as it is often an individual/solitary exercise.

Until recently, little guidance was available to peer reviewers, though, publishers and journals have started developing resources for novice and potential peer reviewers (Pierson 2011). The availability of relatively limited information about the peer review process deters authors' and reviewers' ability and willingness to be involved in the process. An awareness of the peer review process may help authors understand the process, and expectations better and therefore, may alleviate their anxiety and facilitate preparation of appropriate quality manuscripts. Experienced authors will be well aware that not every manuscript is accepted and that some journals have very low publication rates. For example, the *Journal of Advanced Nursing* (one of the present authors is an editor) receives approximately 1,400 manuscripts annually and publishes fewer than 20% of them. The *Journal of the American Medical Association* (*JAMA*) receives over 5000 manuscripts annually and publishes fewer than 5% of them (Personal communication from Howard Bauchner, Editor in‐Chief *JAMA*). Such knowledge may also help authors and readers to become involved in the peer review process. This article aims to provide an overview of the peer review process for authors, novice peer reviewers and those who may have an interest in becoming a peer reviewer. Various types of peer review, selection of peer reviewers, the role of peer review, and issues associated with peer review are explored.

## Background to peer review

Peer review lies at the core of science and academic life (Kearney & Freda 2005, Henly & Dougherty 2009). It is an established component of the publication process, professional practice and the academic reward system (Lee *et al*. 2013). The process involves checking or evaluating the scholarly work by a group of experts in the same discipline. The process is used by academic institutions, funding bodies and publishers to identify strengths, weaknesses and the potential to be published of a proposed piece of work (Pierson 2011). It is an essential element of the publication process that purports to ensure quality and excellence in papers published in scientific, educational and professional journals (Henly & Dougherty 2009). The history of editorial review extends over 200 years (Kronick 1990, Rennie 2003); however, the practice of peer review in its current form only developed in the 19th century (Fyfe 2015) and since 1967 peer review has become the norm. It is now considered a gold standard process that not only helps journals to judge manuscripts, but also acts as a criterion to judge the journals (Bordage & Caelleigh 2001). Before the introduction of peer review, the majority of editors of academic and scientific journals were generalists. After World War II, medical and technological advancement and changes made it impossible for generalist editors to judge papers requiring specialist knowledge. Therefore, it was considered necessary to seek the assistance of expert content specialists to assist in the process of reviewing (Christenbery 2011). Since then peer review has become an integral part of the publication process.

## Utility of peer review

There are many beneficiaries of the peer review process and these include authors, editors and publishers, peer reviewers, disciplines and society. The process provides authors with an opportunity to improve the quality and clarity of their manuscript. Publishing in a peer reviewed journal is considered prestigious. Comments provided by the reviewers guide and help the journal's editor and editorial staff to identify acceptable or substandard manuscripts (Christenbery 2011). Editors rely on the peer review system to inform the choices they make among the many manuscripts competing for the few places available for publication (Broome *et al*. 2010, Lipworth *et al*. 2011).

The peer review process is also useful for peer reviewers themselves, as it helps them develop knowledge and expertise in their specific field. Acting as a peer reviewer may also be recognized as an example of ‘contribution to the profession’ in individual performance reviews (Pierson 2011). ‘The peer review process can also affect society at large when a social policy implication is suggested or inferred from the published manuscript’ (Hojat *et al*. 2003, p. 76). In addition, publication of well written, methodologically sound and well informed research and scholarly papers help professions such as nursing to develop.

## Types of peer review

There are, essentially, two types of peer review: closed and open. The former is more common, but the latter is becoming more popular and authors and reviewers encounter both types of reviews. Closed review has two variants – as will be explained – and we are now seeing post‐publication review (PPPR) in some journals. Each method has its own advantages and disadvantages as specified in Table 1.

**Table 1:** Characteristics of various peer review methods

	Characteristics	Advantages	Disadvantages
Closed peer review			
Single blind	Reviewers aware of authors identity and affiliation Authors unaware of reviewers identity and affiliation	Reviewer anonymity is ensured, therefore they can give honest feedback No risk of intimidation from authors	Reviewers may give harsh comments or give negative feedback The reviewer may delay feedback to delay the publication of manuscript in case they are interested in publishing on the same topic
Double blind	Neither authors nor reviewers are aware of each other identity or affiliation	Reviewer anonymity is ensured, therefore they can give honest feedback No risk of intimidation from authors The manuscript is judged on its quality and content rather than author	Reviewers may give harsh comments or give negative feedback Reviewers may still be able to identify the author in specialist areas
Open peer review			
Open	Authors and reviewers are aware of each other's identity and affiliation	Reviewers are more tactful and constructive while giving feedback Reviewers are more rigorous as their name appears in the published article.	May make the reviewer fearful leading to a less honest and less critical review Reviewers can be intimidated or threatened
Post‐Publication Peer Review (PPPR)			
Primary PPR	A manuscript is published after initial editorial checks. Invited reviewers are invited to review the article. Authors can revise their manuscript and revisions are published When article passes peer review, it in indexed in databases such as Pub Med, Scopus etc.	A wider group of people is able to comment on the paper Transparent Reviewers can be more rigorous, tactful and constructive as their name is published alongside article	People can be unnecessarily harsh or negative People may comment on how the study should have been done rather than looking at the strengths and limitations of the approach used
Secondary PPPR	A manuscript is published after initial editorial checks. Reviewers volunteer to review Various publishers require various criteria. for instance, some require reviewers to have at least 5 publications of their own; other requires reviewers to be registered on databases Authors can revise their manuscript and revisions are published When article passes peer review, it in indexed in databases such as Pub Med, Scopus etc.	A wider group of people is able to comment on the paper Transparent Reviewers can be more rigorous, tactful and constructive as their name is published alongside article	People can be unnecessarily harsh or negative People may comment on how the study should have been done rather than looking at the strengths and limitations of the approach used
Other form of PPPR	People comment on already published articles through blogs, twitter and using other social media	A wider group of people is able to comment on the paper Transparent Reviewers can be more rigorous, tactful and constructive as their name is published alongside article	In experienced reviewers and those with lack of subject knowledge can post irrelevant and unhelpful comments

### Closed peer review

Closed peer review is a system where either the identities of at least one of the parties in the review process – usually the reviewers – are not disclosed. Closed review works in two ways: single blind and double blind. In *single blind review*, the author is not aware of the reviewers' identities. However, the reviewers are aware of the authors' identities, affiliations and credentials. It is the most common approach used in the majority of academic and scientific journals, especially biomedical journals (Kearney & Freda 2005). The method is criticized for several flaws such as the possibility of reviewer bias as the reviewer is not blinded to the details of the authors. The method could be considered unfair on the grounds that the manuscript is the intellectual property of the author (Dividoff & DeAngelis 2001) and, therefore, should be reviewed openly and not secretly (Smith 1999). Some believe that the single blind review gives the reviewers an opportunity to be harsh to the authors as they feel assured that the authors will not be able to identify them. In addition, reviewers working in the same field may delay the feedback to delay publication, if they themselves are thinking of publishing on the same topic. Despite this criticism, single blind peer review remains a commonly used method.

*Double blind review* is also commonly used by many professional biomedical journals (Kearney & Freda 2005, Baggs *et al*. 2008). Nearly all (95%) nursing journals follow this approach (Kearney & Freda 2005). In this approach, the authors and reviewers are not aware of each other's identities and institutional affiliations. Proponents of double‐blind review maintain that this approach eliminates chances of bias in the manuscript review process; whereas, opponents believe that such blinding does not improve the quality of the review (van Rooyen *et al*. 1998, Shea *et al*. 2001). Evidence suggest that, despite double blinding, reviewers may still be able recognize authors through other markers such as writing style, subject matter and self‐citation. Like the single blind review, there is a chance that the reviewers may be unnecessarily critical while giving feedback to the authors.

### Open peer review

In contrast to the closed review, open peer review is a system where authors and reviewer are known to each other throughout the process. Many major journals such as the British Medical Journal (BMJ) encourage this approach. In an open review, authors and reviewers' names may be published alongside each other with an option to publish reviewers' reports alongside. Proponents believe that this is a better approach as nothing is done in secret and the authors' intellectual property rights are respected (Dividoff & DeAngelis 2001). The approach may also act as a regulatory mechanism for the reviewers whom ‘will produce better work and avoid offhand, careless or rude comments when their identity is known’ (Ware 2008, p. 6). Reviewers are recognized for their contribution as their names are published in the journal. Opponents, however, maintain that open review may lead to less honest, less critical and less rigorous review by the reviewers who may fear revenge. Opponents believe that knowing the authors' identity, reputation and institutional affiliation may affect the review process and contribute to a biased decision. We also consider it possible that some reviewers may be overly critical with the intention of appearing to be very rigorous to their peers. Open reviewing recently received some criticism following an incident involving the open access online journal PloS One (Bernstein 2015). The case involved some sexist remarks from a reviewer towards an author advising her to work with male colleagues who were, ostensibly, more successful. This was made possible by dint of the fact that the reviewer could identify the author and her gender due to the open review system. The reviewer and the editor who allowed the comments to be passed on to the author are no longer associated with the journal.

### Other forms of peer review

Hunter (2012, p. 1) states ‘Peer review is broken’ and she continues to explain that, from the author's perspective: ‘Peer review is slow; it delays publication. It's almost always secret; authors do not know who is reviewing their work – perhaps an ally but, equally, perhaps a competitor'. However, more recently, advances in the electronic publishing technology (Ware 2008) have enabled the development of another form of review called ‘post‐publication peer review’ (PPPR), which means that the review is performed once the article is already published. Initially, PPPR was only generally acceptable as a supplement to the peer review process and not as a sole process (Ware 2008) but is becoming more mainstream and, for example, the blog The Future of Scientific Publishing (https://futureofscipub.wordpress.com/open-post-publication-peer-review/; accessed 8 December 2015) advocates more post‐publication reviewing as a form of scrutiny of papers which are in the public domain and, moreover, advocates and open system of review. By some this has been seen as a response to the: ‘urgent need to reform the way in which authors, editors, and publishers conduct the first line of quality control, the peer review’ (Teixeira da Silva & Dobránski 2015, p.1). PPPR can take two forms ‘primary PPPR’ or ‘secondary PPPR’. In primary PPR, an unreviewed article is published after initial editorial checks. It can then be reviewed by formally invited reviewers, as practiced by F1000Research and Copernicus journals (Amsen 2014) who describe their process as ‘publish then filter’ (Hunter 2012). In secondary PPPR, the aricle is published after initial editorial checks but it is available for review by voluntary reviewers. In both cases, the article is altered by the authors on the basis of the PPPR comments and, essentially, evolves towards a published peer reviewed article. Thus, PPPR – of whatever form – complements traditional peer review and ‘allows for the continuous improvement and strengthening of the quality of science publishing’ (Teixeira da Silva & Dobránski 2015, p.1) and now has some prominent supporters, including Richard Smith (2015), the former Editor of the *BMJ*.

In terms of accelerating the peer review process, regardless of the outcome, Kriegeskorte (2012) indicates that the PPPR system essentially merges the ‘review and reception’, or publication, of articles. He envisages the literature being accessed by web‐portals which take readers directly to articles based on subject material rather than through journals or journal webpages, admittedly something that is already evident, and thus facilitating the process of review and the reputation of individual articles rather than journals. Kriegeskorte (2012) sees this as an alternative to potentially good articles being rejected on submission and also the rapid, and possibly undeserved, reputation that some articles gain. In Kriegeskorte's words (p. 7) ‘important papers will accumulate a solid set of evaluations and bubble up in the process – some of them rapidly, others after years’. Naturally, some ‘quality control’ of reviewers is exercised as some publishers require peer reviewers to meet certain criteria. For instance, Science Open requires a reviewer to have at least five articles published in their ORCiD profile. However, at Winnower, any registered user can review a published article and leave their comment (Amsen 2014). Alternatively, commenting on published articles via blogs or other third party sites is always possible.

An informal system of PPPR has always existed and this has been facilitated by the recent major advances in electronic publishing and by the near universality of journals being published online. The rise of online social media and networking is now facilitating, in turn, a steady stream of comment on publications. Authors increasingly ‘get their retaliation in first’ by eking out results and manuscripts through social media platforms such as blogs and microblogs – most specifically, Twitter – whereby an exchange of views can take place in advance, even, of a refereed article. In addition, some journals publish open access; some exclusively and some offering the facility to publish articles open access for a fee called an APC (article processing charge). Even if the content is not freely available, academics have easy access to most scientific publications through their university libraries via gateways such as ATHENS. This means that, with the use of online early publication, by many publishers, of articles before they are serialized and with the immediate posting of articles by some online open access publishers such as BioMed Central, that academics have access to a steady stream of articles in their field. Where scientific literature may not be as freely available, for example, in some developing countries and to those working outside academic publishers do take steps to increase ease of access to their work through specific deals and, of course, it is always open to any academic to request an offprint (hard copy or electronic) directly from authors.

Finally, and very recently, is the advent of the website PubPeer which explicitly exists to provide anonymous post‐publication review of published, refereed, articles. As explained by Watson (2016), PubPeer is in its infancy, but growing and has received some negative press as in the description of promoting ‘vigilante science’.

## Selection of peer reviewers

Reviewers are usually people who have published on the same topic (Brazeau *et al*. 2008) and selection of the reviewer is an important task that is normally carried out by the editor of the journal. Editors identify and invite suitable, experienced and interested people in the subject matter or relevant field by using the key words authors (peer review) have used in the past. Many journals use a bank of established and regular reviewers, but some use the keywords to identify individuals via search engines and databases, for example, ResearcherID. Some journals ask the authors to name reviewers and one study (Kowalczuk *et al*. 2015) suggest that, while this has little effect on the quality of reviews, it does lead to higher recommendations to accept manuscripts. However, the process of authors suggesting reviewers has led to some scandals related to fabricated peer reviews (Barbash 2015, Moylan 2015) and some journals are no longer using this process. In some journals, authors can also indicate individuals they would not wish to review their manuscripts. The editors may also invite authors to become subsequent reviewers, sometimes by asking them to provide their *Curriculum vitae* (Evans *et al*. 1993) or on the basis of particular qualifications (e.g. a PhD) and a publication track record in peer reviewed journals. The method of selecting the reviewer does not, necessarily, affect the quality of the review as individuals are different and, therefore, their interpretation, views and methods of review will, in any case, vary. However, contrary to what might be expected, it has been demonstrated that emerging academics are usually better reviewers as they provide comprehensive and thorough feedback (Evans *et al*. 1993, van Rooyen *et al*. 1998). Evidence also identified no improvement in the quality of review with academic seniority or gender (Gilbert *et al*. 1994, Fox *et al*. 2016).

## Role of peer reviewers

Reviewers contribute to the development of the knowledge base of any profession, such as nursing, by giving their valuable time to review manuscripts (Dipboye 2006, Pierson 2011). Reviewers are volunteers and rarely receive any monetary compensation for their role (Relman & Angell 1989). The role of a reviewer is very important, yet a challenging and complex professional activity. To be a good reviewer requires theoretical, methodological and practical knowledge and an ability to apply that knowledge when evaluating a manuscript and writing constructive feedback to help the author improve the quality of their manuscript (Lovejoy *et al*. 2011). In addition, reviewers' feedback helps the editor to make a decision about the manuscript (Broome *et al*. 2010). Acting as a peer reviewer is useful for an individual academic, as it helps them to develop their subject knowledge, analytical abilities and skills required to provide constructive feedback. The activity is usually recorded on their curriculum vitae and thus can be recognized in performance appraisal and progression. There are various reasons why reviewers choose to review manuscripts. These include a desire to play their part as a member of the academic community, improve their reputation and career progression (Ware 2008) and increase their knowledge and understanding of their subject. Other common factors that may encourage academics to act as peer reviewer include the inducement of getting a free or reduced subscription to the journal, acknowledgement in journals and payment in kind (Ware 2008). The reviewers have to adhere to certain principles of the review as advocated by the Committee on Publication Ethics (2013) and academic journals. These are summarized in Table 2.

**Table 2:** Principles of Peer Review recommended by Committee on Publication Ethics (2013)

Principles
Only agree to review manuscripts that they have subject expertise in Review manuscript in a timely manner Respect confidentiality of the review process Do not use information obtained during peer review process for own or anyone else advantage or disadvantage Declare conflict of interest, if any Do not let author's characteristics (age, gender, and nationality, religious or political beliefs) influence review Provide constructive and objective feedback about the manuscript under review

## Issues with peer review

As already indicated, the peer review process is criticized by many academics who believe ‘…it is ineffective, largely a lottery, anti‐innovatory, slow, expensive, wasteful of scientific time, inefficient, easily abused, prone to bias, unable to detect fraud and irrelevant’ (Smith 2015). Some believe that various flaws and problems in the peer review process may affect the quality of reviews and, thereby, the quality of publications. These flaws include: slowness of the publication processes; negative impact on authors; poor preparation and training of reviewers; variable review requirements; ineffectiveness of peer review; and biases in peer review. We believe, these issues are relevant to all forms of peer review, although, some may be more relevant to some forms of peer review than others.

### Peer review slows the publication process

There is a perception that peer review may slow the process of publication. ‘…the original purpose of peer review was to ration access to resources for scholarly exposure. Nowadays, however, exposure is not a scarce resource, since publications can be made available electronically, essentially free of cost. The question, therefore, is one of quality control and we do not know how much refereeing the scholarly market actually wants’ (The British Academy 2007, p. 11). However, peer review is a quality control mechanism which, despite contributing to slowness of procedures, enhances the quality of the publication. In addition, most journals – these days – not only specify a date when a review is due, but also send reminders (a week before the review is due; on the due date) to reviewers to remind them to complete and submit their review timely. This approach is very useful as it helps reviewers to complete their review in time.

### Negative impact on authors

Undergoing peer review can be a negative experience for some authors due to insensitive and irresponsible behaviour of some reviewers who may not read the manuscript, provide irrelevant comments or feedback, and use the opportunity to promote their work or make negative and malicious comments (Smith 2015). However, development and communication of appropriate practice guidelines and principles of peer review may help overcome such issues. In addition, the journal editors can play a very important role and may be able to intervene in such situation by discussing the issues with the reviewer. This issue may have more impact in the context of post‐publication peer review. Publicly available harsh, unnecessary, negative and insensitive comments can be detrimental to author's rapport and may have an impact on their confidence and ability to write in future.

### Poor reviewer preparation

Formal training and preparation may help reviewers develop appropriate review skills, but is often not widely available. The process itself is not easy to learn (Provenzale & Stanley 2006) and educational programmes do not prepare postgraduate students for the role of peer reviewer (Eastwood 2000). This, in turn, affects the confidence and ability of reviewers who may only learn the art of reviewing through trial and error. New reviewers usually do not have any training or awareness about how to review a paper. A reviewer may not have any mentorship or any experience of reviewing someone else's work. This issue can be overcome by ensuring that postgraduate students, doctoral and post‐doctoral academics are provided with appropriate training and guidance to develop their review and feedback skills (The British Academy 2007, House of Commons Science and Technology Committee 2011). One strategy may be that postgraduate students and emerging academics should be invited to review manuscripts as a third reviewer. Appropriate mentorship and guidance can be provided by introducing a buddy system where novice reviewers are ‘buddied’ with experienced reviewers. In either of the cases above, this needs to be done with the permission of the journal and declared and some journals ask for this as a specific declaration when reviews are submitted. This may help novice reviewers to develop reviewing skills and knowledge. Presently, very few journals give reviewers access to other reviewers' comments. Nevertheless, giving reviewers access to other reviewers comments about the same manuscript can also be a useful way of helping reviewers improve their knowledge and skills (House of Commons Science and Technology Committee 2011). As manuscripts are now reviewed electronically, providing access to other reviewers' comments and feedback is fairly straightforward and hassle‐free.

### Variable review requirements

There is a wide variation in the review requirements and expectations among different journals. Recently, various publishers and journals have started to develop guidelines to help reviewers understand the expectations. Some journals are very prescriptive and expect strict compliance by the reviewers, while others may be less specific about their expectations. Although it is important to provide some guidance about review and communicate expectation to ensure consistency in review, too much prescription may limit the reviewer's ability to critically assess and feedback on strengths and areas of improvement of a manuscript. Again providing appropriate guidance, mentorship opportunities and sharing of fellow reviewer's reports can help reviewers identify their own style of review and develop confidence and ability to provide constructive feedback.

### Ineffectiveness of peer review

Research examining effectiveness of peer review is still limited (Patel 2014). The lack of research supporting or negating the effectiveness of peer review contributes to ambiguity about the effectiveness of peer review and fuels the criticism against peer review (Jefferson *et al*. 2002, Ware 2008, Patel 2014). Some researchers consider peer review as an unreliable method of quality assurance and error detection (Godlee *et al*. 1998, Patel 2014). They believe that reviewing by two reviewers is insufficient to identify issues with the manuscript. The authors maintain that to make the peer review process reliable and comparable, an editor is required to have a minimum of six reviewers, whereas generally, it is often difficult to identify two or three reviewers to review a paper (Rothwell & Martyn 2000, Ware 2008). It should also be recognized that peer review is not a scientific process; it is a process based on people and the judgements they make. People differ in their expertise, opinions and experience and, therefore, their opinion or feedback about same manuscript can differ. In addition, reviewers do not make the decisions about which manuscript to accept or reject, but only provide their view on a manuscript, which aids the editors in making a decision.

### Peer review and bias

The peer review process cannot be free from bias; bias can only be minimized. Generally a single blind review is criticized for the risk of bias. However, the effectiveness of the blinding process itself is questionable (Kearney & Freda 2005, Baggs *et al*. 2008, Ware 2008). Another flaw of the peer review system is the biased decisions of the peer reviewers. Evidence suggests that reviewers tend to accept papers that provide confirmatory results and reject those that do not confirm established theories (Mahoney 1977). Similarly, peer reviewers tend to accept studies that offer positive results and reject those that report negative results. This issue is referred to as ‘file drawer problem’ (Rosenthal 1979 p. 638) as the research with negative results due to non‐acceptance remain in the file drawer of the researcher and are not disseminated to the wider community. Some researchers have even mentioned that peer review works against innovative studies (Armstrong 1996, Hojat *et al*. 2003, Lee, *et al*.^2013^), a point reinforced recently by the former Editor of the BMJ (Smith 2015). Reviews can also be influenced by the characteristics of authors (gender, political or religious affiliation, institutional affiliation, nationality, country of origin) (Smith 2015, Fox *et al*. 2016) and whether they are identified by the editor or proposed by the author (Kowalczuk *et al*. 2015). These issues can be minimized by ensuring reviewers are aware of and adhere to ethical principles of review.

Despite various issues, the usefulness of the peer review process cannot be overlooked. The process of peer review, mainly in publishing but also in other aspects of academic life is regularly discussed (Fyfe 2015, Smith 2015). The process recently came under the scrutiny of the British government (House of Commons Science and Technology Committee 2011) and other bodies (Watson 2012) after some accusations about biased publishing in the field of climate science. The scrutiny was in‐depth and prolonged, but the conclusion was that the peer review system in it various manifestations were far from perfect, but that it was the best we had and should continue.

## Conclusion

It is essential to remember that peer reviewing is a voluntary activity, which means that the reviewers are not paid for their work and often complete reviews in their own time. While contributing to reviewing processes is a professional and moral obligation of any author whose work has undergone peer review (Priem & Rasheed 2006), it is important to make this activity as rewarding and developmental as possible. Recognizing reviewers for their work by publishing their names in the journal or providing them with awards and recognition certificates can be a useful strategy. More recently, various publishers and journals have started using these strategies to recognize the reviewers' contribution. Such strategies may be useful and may increase the motivation of reviewers and, in turn, may enhance quality of review by reviewers.

Peer review is one of various mechanisms used to ensure the quality of publications in academic journals. It helps authors, journal editors and the reviewer themselves. It is a process that is unlikely to be eliminated from the publication process. All forms of peer review have their own strengths and weaknesses. To make the process more effective and useful, it is important to develop peer review skills, especially, among postgraduate students. There should be published guidelines and help for novice peer reviewers. Mentoring new reviewers and sharing the feedback of different reviewers can help new reviewers. More research is needed to determine the effectiveness of the peer review process.

## Conflict of interest

None.

## Author contributions

All authors have agreed on the final version and meet at least one of the following criteria [recommended by the ICMJE (http://www.icmje.org/recommendations/)]:

- substantial contributions to conception and design, acquisition of data, or analysis and interpretation of data;
- drafting the article or revising it critically for important intellectual content.
